# Understanding Thiel Embalming in Pig Kidneys to Develop a New Circulation Model

**DOI:** 10.1371/journal.pone.0120114

**Published:** 2015-03-25

**Authors:** Wouter Willaert, Marie De Vos, Tom Van Hoof, Louke Delrue, Piet Pattyn, Katharina D’Herde

**Affiliations:** 1 Department of Gastrointestinal Surgery, Ghent University Hospital, Ghent, Belgium; 2 Department of Basic Medical Sciences, Ghent University Hospital, Ghent, Belgium; 3 Department of Radiology and Medical Imaging, Ghent University Hospital, Ghent, Belgium; The University of Tokyo, JAPAN

## Abstract

The quality of tissue preservation in Thiel embalmed bodies varies. Research on the administered embalming volume and its vascular distribution may elucidate one of the mechanisms of tissue preservation and allow for new applications of Thiel embalming. Vascular embalming with (group 1, n = 15) or without (group 2, n = 20) contrast agent was initiated in pig kidneys. The distribution of Thiel embalming solution in group 1 was visualized using computed tomography. The kidneys in both groups were then immersed in concentrated salt solutions to reduce their weight and volume. Afterwards, to mimic a lifelike circulation in the vessels, group 2 underwent pump-driven reperfusion for 120 minutes with either paraffinum perliquidum or diluted polyethylene glycol. The circulation was imaged with computed tomography. All of the kidneys were adequately preserved. The embalming solution spread diffusely in the kidney, but fluid accumulation was present. Subsequent immersion in concentrated salt solutions reduced weight (*P* < 0.01) and volume (*P* < 0.01). Reperfusion for 120 minutes was established in group 2. Paraffinum perliquidum filled both major vessels and renal tissue, whereas diluted polyethylene glycol spread widely in the kidney. There were no increases in weight (*P* = 0.26) and volume (*P* = 0.79); and pressure further decreased (*P* = 0.032) after more than 60 minutes of reperfusion with paraffinum perliquidum, whereas there were increases in weight (*P* = 0.005), volume (*P* = 0.032) and pressure (*P* < 0.0001) after reperfusion with diluted polyethylene glycol. Arterial embalming of kidneys results in successful preservation due to complete parenchymatous spreading. More research is needed to determine whether other factors affect embalming quality. Dehydration is an effective method to regain the organs’ initial status. Prolonged vascular reperfusion with paraffinum perliquidum can be established in this model without increases in weight, volume and pressure.

## Introduction

In 1992, Thiel reported a new soft embalming technique, which presently consists of vascular perfusion followed by immersion in a bath for at least two months [1, 2]. This technique is exceptional because the colour, consistency and transparency of the tissues are very well preserved. Moreover, preservation is long-lasting and no harmful substances are released into the environment [1]. This soft-fix embalming technique provides a more realistic tool for surgical training when compared with formalin-embalmed cadavers [3, 4]. In addition, the bodies are more realistic than fresh-frozen material, which suffers from postmortem rigidity and putrefaction [5, 6]. As a result, Thiel cadavers are increasingly used for dissection courses, research purposes and training in several disciplines [3, 5–14].

Today, several issues concerning the Thiel embalming procedure are not standardized or remain unknown and unresolved. Consequently, there is not a standard technique to ensure high quality tissue preservation. Thiel recommends a vascular embalming volume of 15.8 L for one complete cadaver [1]. This volume provides a sufficient distribution in the body but is probably too abundant because we often observe the escape of embalming fluid via the ears and nose. Presumably, large amounts of fluid in the capillaries extravasate and accumulate in the extravascular tissues before eventually leaving the body. Thiel embalmed cadavers usually look slightly bloated [5]. Later, the fluid gradually drains out, and the body returns to its pre-embalming appearance. Remarkably, this swelling, which suggests diffuse capillary spreading of the embalming product, is often not associated with uniform, high quality preservation of the body.

Several issues must be resolved to perform solid embalming with Thiel embalming fluid. The vascular perfusion properties of Thiel embalming fluid have not yet been explored, which may explain why high quality tissue preservation is not always observed. Therefore, in this study, a kidney model was used to assess if incomplete vascular distribution of a fixed embalming volume is the causative factor for the observed variability in tissue preservation. Because embalming causes tissue swelling and deformation, an osmotic dehydration method was tested in Thiel embalmed kidneys to re-establish their original status in a fast and controlled way. Moreover, as part of a project to develop an ideal surgical training model of reperfusion in the vessels of Thiel embalmed human cadavers, we explored if a continuous pump-driven flow mimicking the circulation of blood can be established in the vessels of the dehydrated Thiel embalmed kidneys.

In this study, we demonstrate that variability in tissue embalming quality cannot be explained by insufficient vascular spreading of a fixed embalming volume. In addition, embalming-induced swelling and deformation can be successfully halted by salt water immersion, which creates ideal circumstances for prolonged reperfusion of the renal vessels with paraffinum perliquidum (PP).

## Materials and Methods

This study was approved by the Committee on the Ethics of Animal Experiments of the University of Ghent, Belgium (approval code: 11/36).

### Experiment 1

Fifteen fresh pig kidneys (group 1) were obtained from the slaughterhouse. Kidneys were chosen because they usually have only one main feeding artery and one draining vein, allowing to easily install a vascular circulation. Initially, the renal artery and vein were identified and the ureter was ligated. The specimens were then weighed and their volumes were measured by immersion in a vessel full of water (Archimedes’ principle). Next, a Quik-Cath II 14-gauge catheter (Baxter, Mayo, Ireland) was placed in the renal artery and connected to a tube, which was placed in a roller pump (Watson-Marlow 520 U, Zwijnaarde, Belgium) that initiated Thiel embalming. The weight of the injected embalming solution was 22.7% of the weight of the dissected kidney. This amount is in accordance with the administration of 18.170 kg of embalming solution (or 15.8 L) to a human cadaver of 80 kg as proposed by Thiel. In addition, the volume of the administered embalming product was measured. Next, a contrast agent (Omnipaque 300, GE Healthcare, Diegem, Belgium) was mixed with the embalming solution. The volume of contrast agent added was 10% of the administered embalming volume.

**Fig 1:**
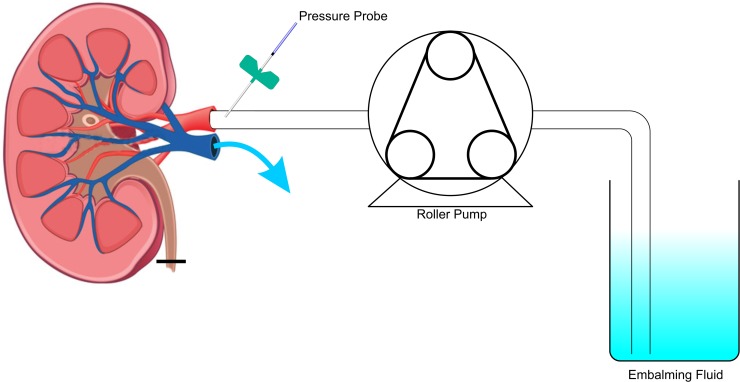
Pressure-controlled embalming of pig kidneys. Thiel embalming fluid is pumped in the renal artery, and a mixture of blood and/or embalming fluid eventually leaves the kidney through the renal vein.

After initiating the embalming procedure, the renal artery was punctured with a BD Insyte-W 22-gauge catheter (BD Vialon, Madrid, Spain) and connected to an ultraminiature fibre optic pressure transducer (Samba 201 CAP, Harvard Apparatus, Cedex, France). The maximum intra-arterial pressure allowed was 65 mmHg, in agreement with the *in vivo* arterial blood pressure of pigs (Fig. 1). The type of venous drainage (transparent, serosanguinous or sanguinous) was assessed at the end of the embalming procedure. Subsequently, the weight, volume and swelling of the kidneys were noted. The vascular spreading of the embalming product was imaged by computed tomography (CT; Somatom Definition Flash, Siemens Healthcare Sector, Forchheim, Germany).

**Fig 2:**
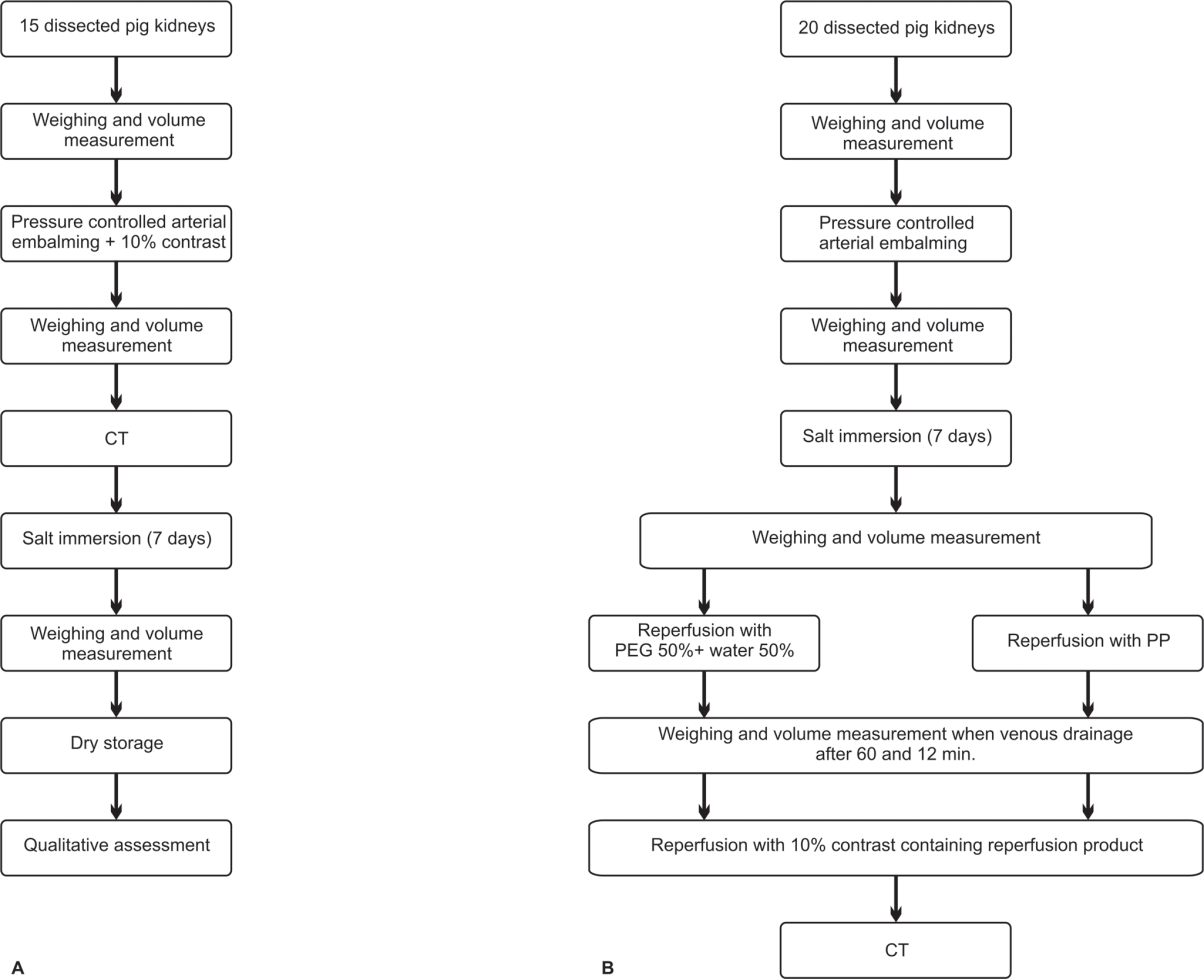
Stepwise illustration of both experiments. (A) Pressure-controlled Thiel embalming followed by immersion in a concentrated salt solution. (B) Reperfusion of Thiel embalmed and dehydrated kidneys with either PP or diluted PEG.

The embalming procedure causes diffuse parenchymatous swelling and changes the macroscopic appearance of the kidney. Next, to lower the weight, each kidney was immersed in a concentrated salt solution of 0.300 kg salt/L tap water for seven days. The amount of salt (kg) was the same as the weight of the embalmed kidney. This caused a movement of superfluous solvent molecules from the kidney into the concentrated salt solution, without interfering with the embalming procedure. After seven days, the weight, volume and appearance (i.e., presence or absence of shrinkage) were noted. The kidneys were then stored in a refrigerator at 9.5°C. After one week, the embalming quality was evaluated in terms of the general appearance, yeast formation and putrefaction. Fig. 2A depicts the design of the experiment schematically. Lastly, the dynamic viscosity of the administered embalming fluid was determined at 25°C with a Micro-Ubbelohde Viscometer (Schott-Geräte, Mainz, Germany).

### Experiment 2

In this experiment, a pump-driven flow was re-established in the renal vascular system. Therefore, twenty fresh pig kidneys (group 2) from the slaughterhouse underwent the same procedures described above, but no contrast agent was added during embalming (Fig. 2B). The dehydration procedure aimed to reduce the pressure on the vessels, facilitating a subsequent vascular reperfusion. The pigs were randomly divided into two groups. In one group, the vessels were reperfused with red PP (i.e., PP containing 43 mg/L Oil Red O [both from Sigma-Aldrich, Bornem, Belgium]). In the other group, the vessels were reperfused with diluted polyethylene glycol (PEG; i.e., 50% PEG 400 [Sigma-Aldrich, Bornem, Belgium] and 50% tap water).

Before establishing the reperfusion, the volumes and weights of the kidneys were measured anew. Next, a reservoir was filled with 100 mL of the allotted perfusate. Subsequently, as described in the first experiment, the 14-gauge arterial catheter was reconnected to a tube that was placed in the pump, and the 22-gauge catheter was connected to the pressure transducer. In this way, a pump-driven, pressure controlled (< 65 mmHg) injection of the allotted perfusate was installed to the renal artery and kidney until there was venous drainage in the reservoir. At that time, both catheters were disconnected and the renal volume and weight were redetermined. After reconnecting the catheters, a closed circulation with the perfusate was re-established, i.e., the venous drainage in the reservoir was pumped into the renal artery again. The procedure was interrupted after 60 and 120 minutes to measure the volumes and weights of the kidneys. After 120 minutes of reperfusion, we reassessed the renal appearance (swelling, pliability, capsular leak and subcapsular collections).

Reperfusion via the artery was restarted after adding contrast agent to the remaining perfusate in the reservoir. The amount of contrast agent added was 10% of the remaining volume of perfusate. In case of reperfusion with red PP, we used black coloured Angiofil (Fumedica AG, Muri, Switzerland). The diluted PEG was mixed with a combination of Omnipaque 300 and 2 cc of 1% methylene blue (Sterop, Brussels, Belgium). Methylene blue was added to colour the transparent diluted PEG. When the contrast containing mixture left the renal vein, the reperfusion was terminated. CT was performed to assess the vascular distribution of both perfusates. In addition, 4 specimens were frozen at −80°C and sectioned to visualize the vascular reperfusion of PP and diluted PEG.

Finally, the effects of embalming, dehydration and vascular reperfusion on tissue morphology were investigated. Therefore, cortical biopsies were taken from a fresh kidney; a Thiel embalmed kidney; a dehydrated Thiel embalmed kidney; and after 120 minutes of vascular reperfusion with PP and diluted PEG. The biopsies were stained with orcein and hematoxylin and eosin.

### Statistical Analysis

Statistical analysis was carried out with SPSS Version 21.0. Comparisons between different groups were performed with the Friedman and Wilcoxon signed-rank test. A *P* value < 0.05 was deemed statistically significant.

## Results

### Experiment 1

**Fig 3:**
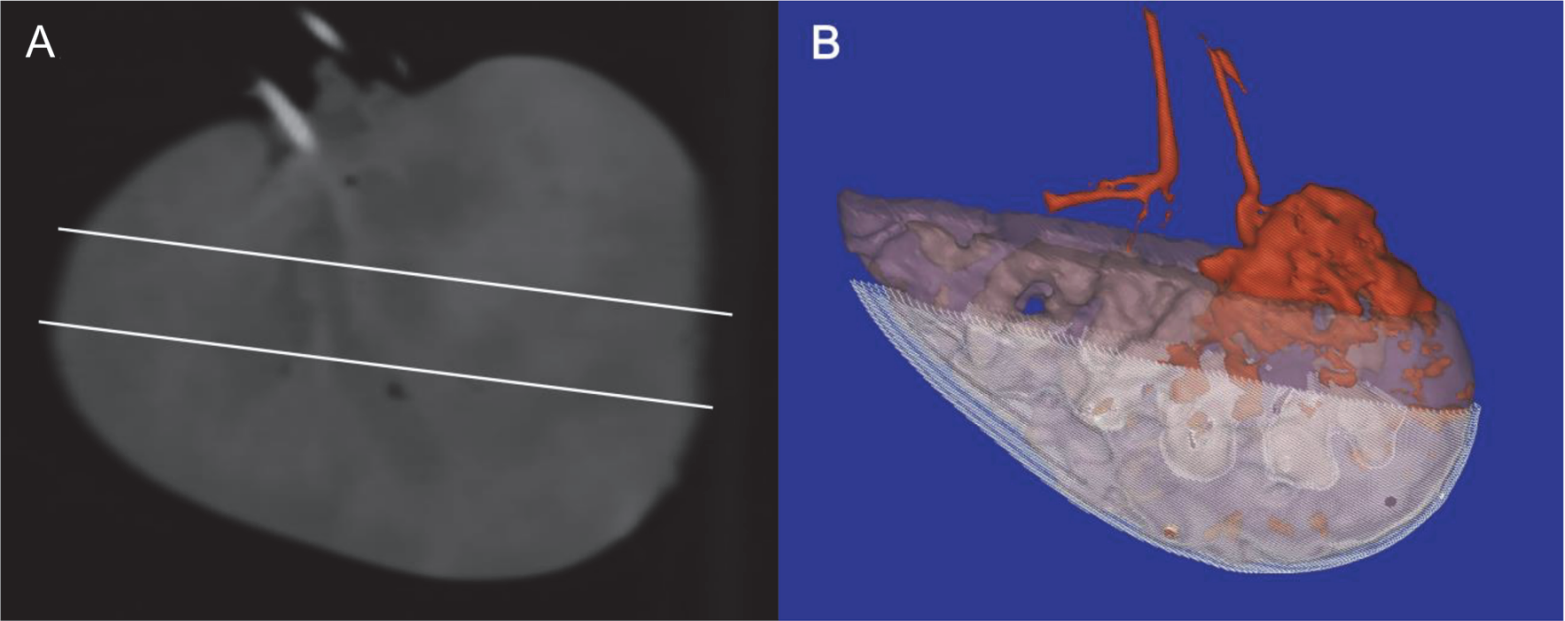
Renal spreading of Thiel embalming fluid. (A) Planar CT image shows three areas of filling (contrast): bright, the main arterial and venous system and the poles-medial border of the kidney; intermediate grey, centrally distributed areas; and darker grey, areas at the core and kidney surface. Two oblique white lines represent virtual slicing through the upper and lower mid-central part of the kidney. (B) Three-dimensional representation of the same kidney; red, main arterial and venous system and one of the poles-medial border areas; transparent purple, centrally distributed areas of the kidney showing intermediate contrast filling; and white polylines, inner core areas (four cone-like structures, calices) and surface of the kidney showing the least contrast filling.

Arterial administration of a fixed volume of contrast-enhanced Thiel embalming solution showed diffuse but variable renal dispersion without zones lacking contrast. In detail, the embalming product filled the renal artery and its major branches, appearing as bright white. There was an overall diffuse intermediate opacification of the renal parenchyma, with local zones of contrast accumulation. The renal surface tended to have less contrast filling, but slightly more than the renal calices. Bright opacification of the draining renal vein was often observed. The renal distribution of the embalming fluid is illustrated in Fig. 3. After embalming, the proportions of kidneys with sanguinous, serosanguinous and transparent venous drainage were 60%, 33.3% and 6.7%, respectively (S1 Dataset). This caused significant increases in weight (*P* = 0.005) and volume (*P* = 0.007), with on macroscopic inspection obvious swelling in 33.3% of cases.

**Fig 4:**
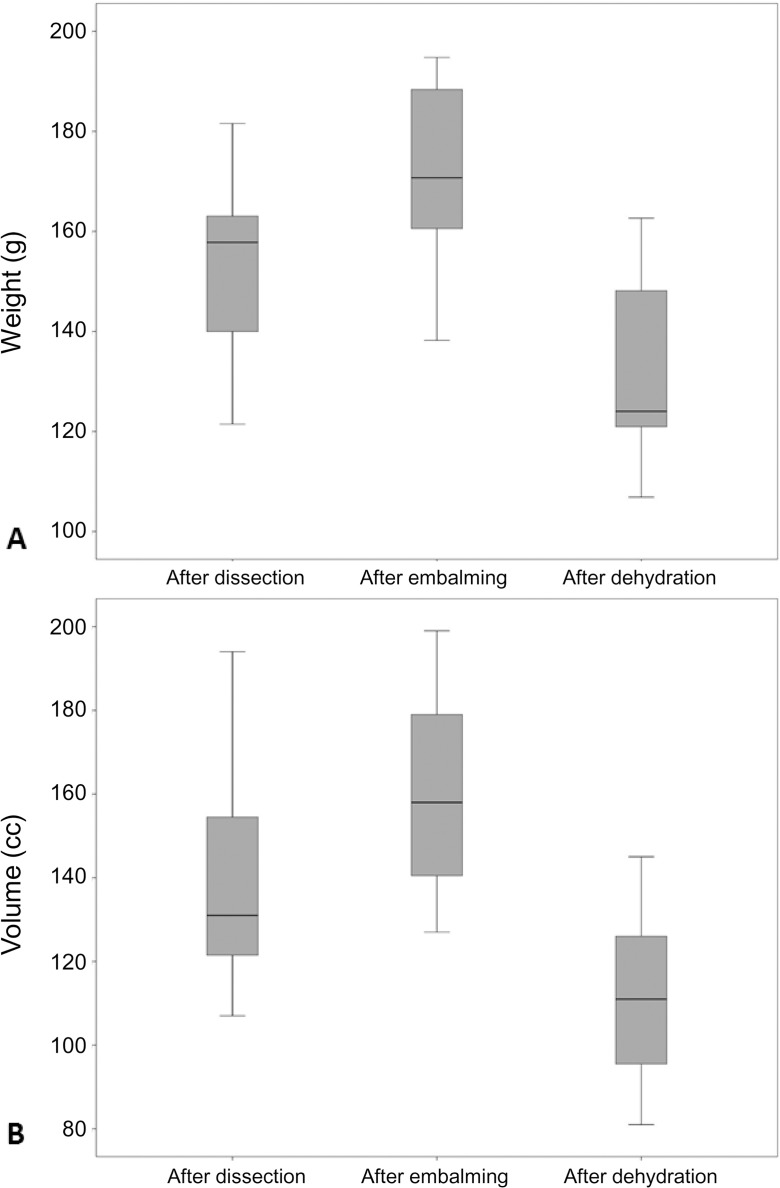
Weight and volume changes in embalmed and dehydrated kidneys. (A) A weight gain occurs after embalming (*P* = 0.005), and a weight loss (*P* < 0.001) occurs following the subsequent dehydration, respectively. The combination of these two procedures results in a significant weight reduction (*P* = 0.007). (B) Swelling occurs after embalming (*P* = 0.007), and volume loss occurs following the subsequent immersion in a concentrated salt solution (*P* < 0.001). The combination of both procedures results in a significant volume reduction (*P* = 0.003).

**Fig 5:**
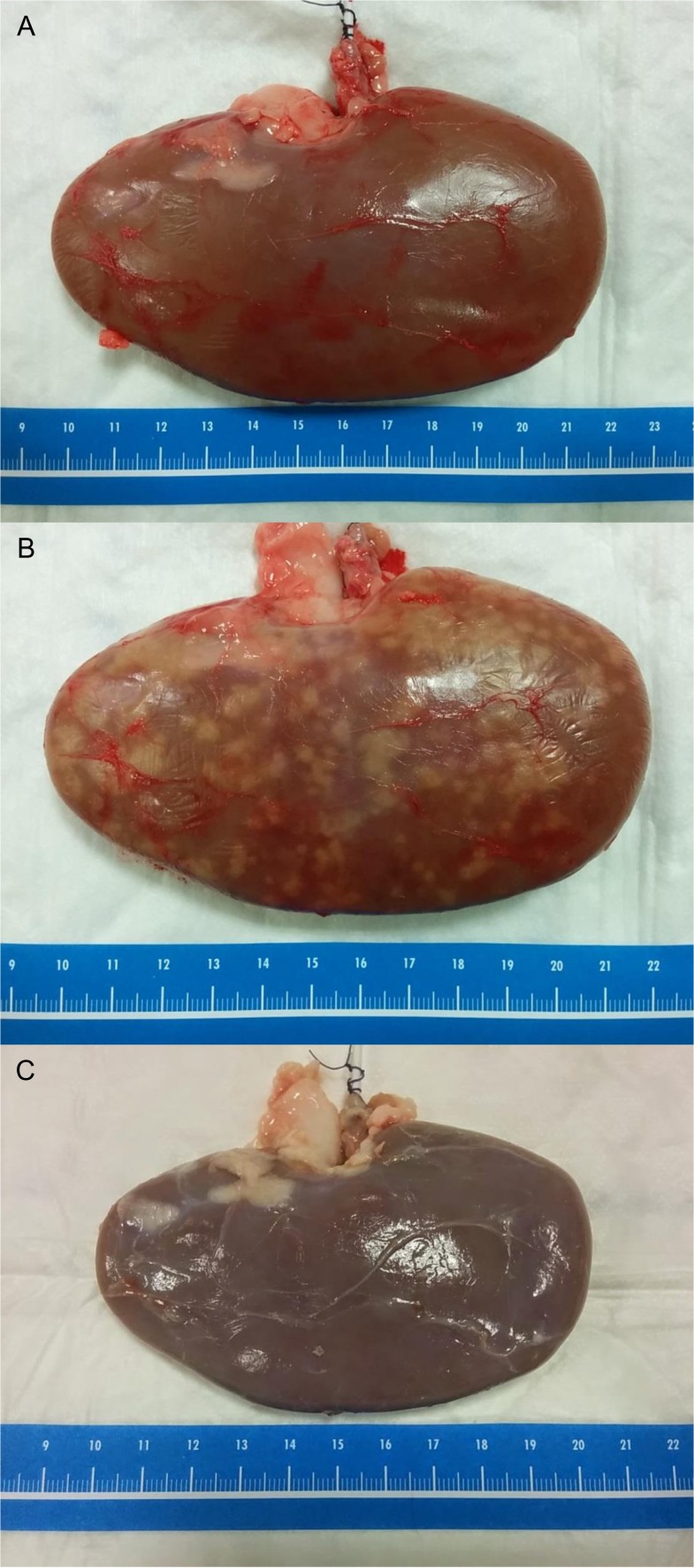
Effect of embalming and dehydration on renal status. (A) Fresh pig kidney. (B) Thiel embalmed pig kidney. Note the swelling and zonal discolouring due to the embalming. (C) Thiel embalmed and dehydrated pig kidney. The organ is shrunken and discoloured, but remains pliable.

Significant weight (*P* < 0.001) and volume loss (*P* < 0.001) were encountered due to the immersion of embalmed kidneys in concentrated salt solutions (S2 Dataset). In particular, dehydration nullified the effect of the embalming procedure. Consequently, the combination of both procedures caused mean decreases in total weight and volume of 16.1% and 26.3%, respectively. As a result, on inspection, the majority of kidneys were shrunken (86.7%). Figs. 4 and 5 show how embalming and subsequent immersion in a concentrated salt solution affected weight and volume.

After one week of refrigeration, excellent preservation was observed in every kidney without yeast formation or putrefaction. The dynamic viscosity of the vascular embalming fluid was 2.17 mPas at 25°C (S3 Dataset).

### Experiment 2

**Fig 6:**
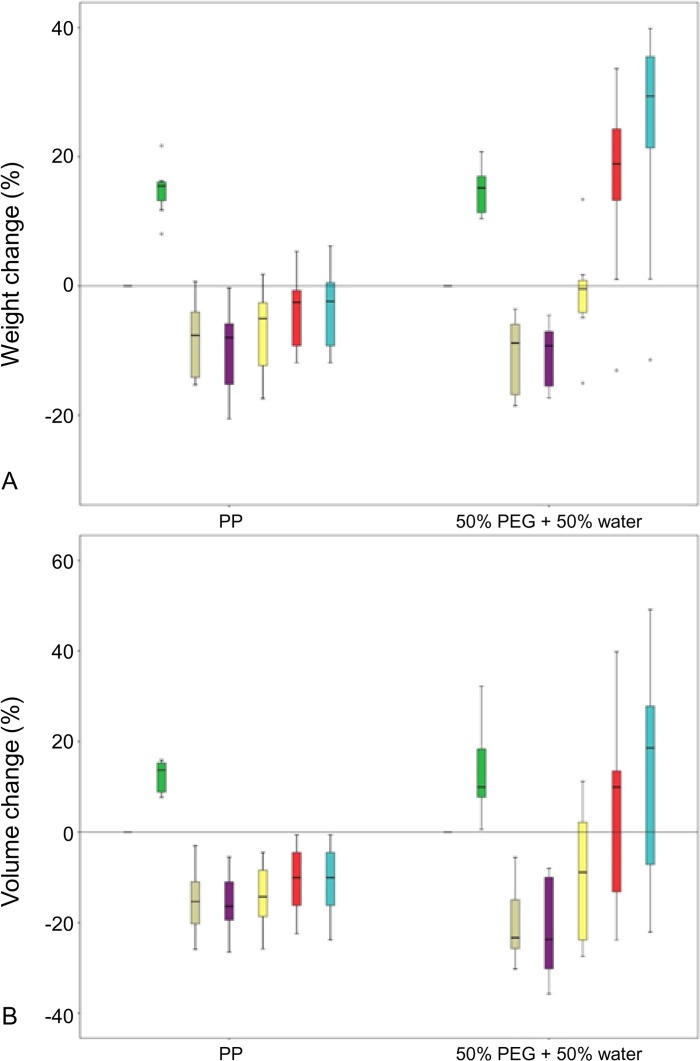
Percent weight and volume change during embalming, dehydration and reperfusion of pig kidneys. (A) Embalming and subsequent dehydration cause a significant weight gain (*P* < 0.0001) and loss (*P* < 0.0001), respectively. Persistent weight gain is observed during initial reperfusion with diluted PEG or PP (both *P* = 0.005), whereas weight gain is not present after more than 60 minutes of reperfusion with PP (*P* = 0.26). In contrast, ongoing weight gain (*P* = 0.005) is noted during reperfusion for more than 60 minutes with diluted PEG. (B) Embalming and subsequent dehydration cause a significant volume increase (*P* < 0.0001) and loss (*P* < 0.0001), respectively. In the beginning, a continuous volume increase is noted in the case of reperfusion with diluted PEG (*P* = 0.005) as well as PP (*P* = 0.007). No further increase is observed after more than 60 minutes of reperfusion with PP (*P* = 0.79), whereas an ongoing volume gain is present in the case of diluted PEG (*P* = 0.032). Weight/volume after embalming = green; after dehydration = brown; at the start of reperfusion = purple; at first venous drainage = yellow; after 60 minutes of reperfusion = red; after 120 minutes of reperfusion = blue.

**Fig 7:**
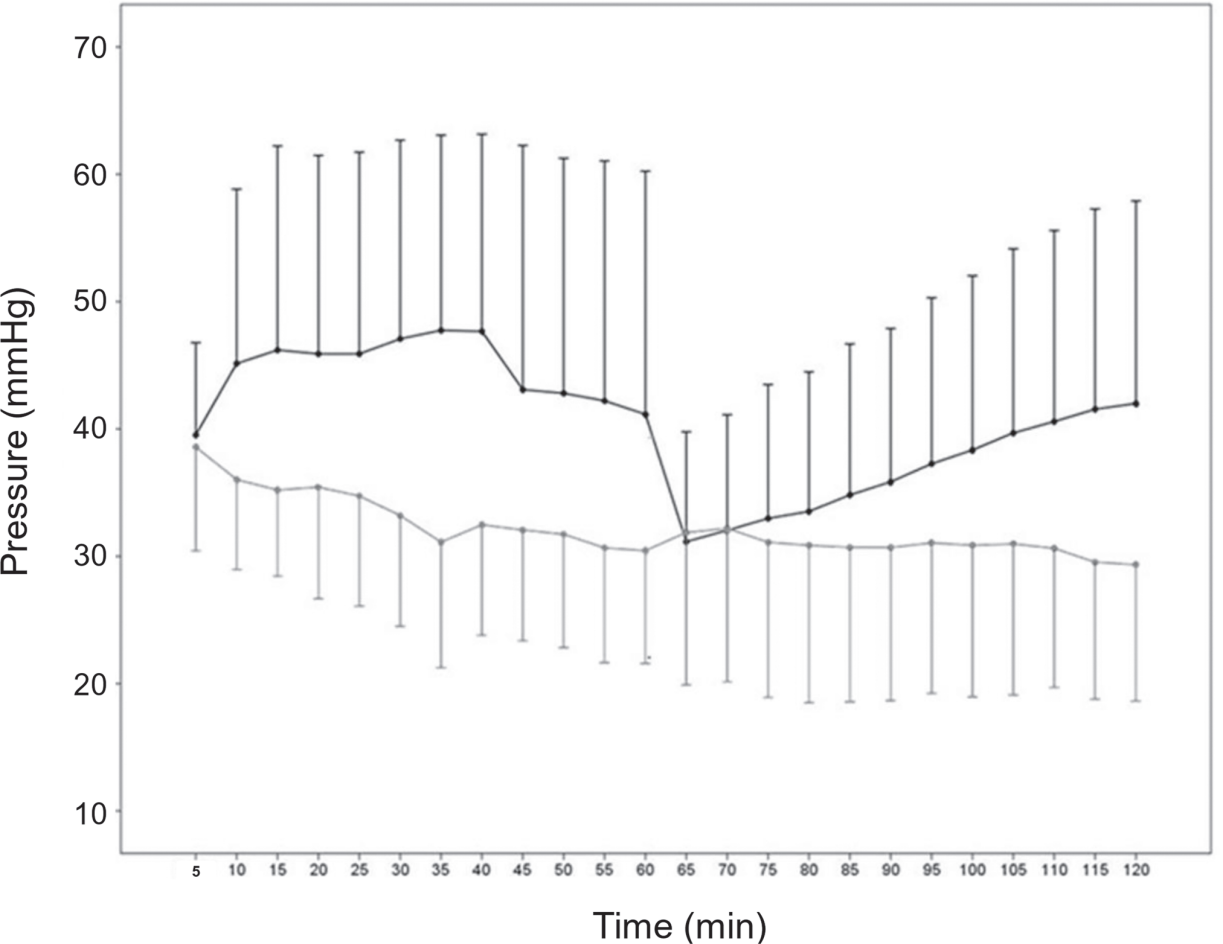
Intra-arterial pressure during renal reperfusion. PP generates lower pressures than diluted PEG (*P* < 0.05). Ongoing pressure decrease is observed during more than 60 minutes of reperfusion with PP (*P* = 0.032), whereas the pressure increases further (*P* < 0.0001) when diluted PEG is used. The generated pressures remain, however, lower than the mean arterial blood pressures in adult pigs. The error bars with 95% confidence intervals are shown.

Each group contained 10 kidneys, which lost significant weight (*P* < 0.0001) and volume (*P* < 0.0001) after embalming and dehydration (S4 and S5 Datasets). In both groups, renal reperfusion for one hour caused a significant weight (both *P* = 0.005) and volume (PP: *P* = 0.007; diluted PEG: *P* = 0.005) increase. Weight (*P* = 0.005) and volume (*P* = 0.032) gain after more than 60 minutes of reperfusion with diluted PEG are substantially greater than with PP (i.e., *P* = 0.26; *P* = 0.79, respectively). Fig. 6 presents the weight and volume changes for the two perfusates during each step of this experiment. PP generated lower intra-arterial pressures during the total reperfusion period (*P* < 0.05). In particular, an initial pressure decrease during the first 60 minutes (*P* < 0.001) persisted over time (*P* = 0.032). In contrast, ongoing pressure increase (*P* < 0.0001) was observed during reperfusion with diluted PEG. Pressure-time curves for both perfusates are illustrated in Fig. 7. Reperfusion of the renal vessels for 120 minutes did not affect the pliability of the organs although obvious swelling was observed in six out of ten kidneys reperfused with diluted PEG (S6 Dataset). After prolonged reperfusion with PP, two limited subcapsular collections (n = 1) and a few capsular drops (n = 1) were observed.

**Fig 8:**
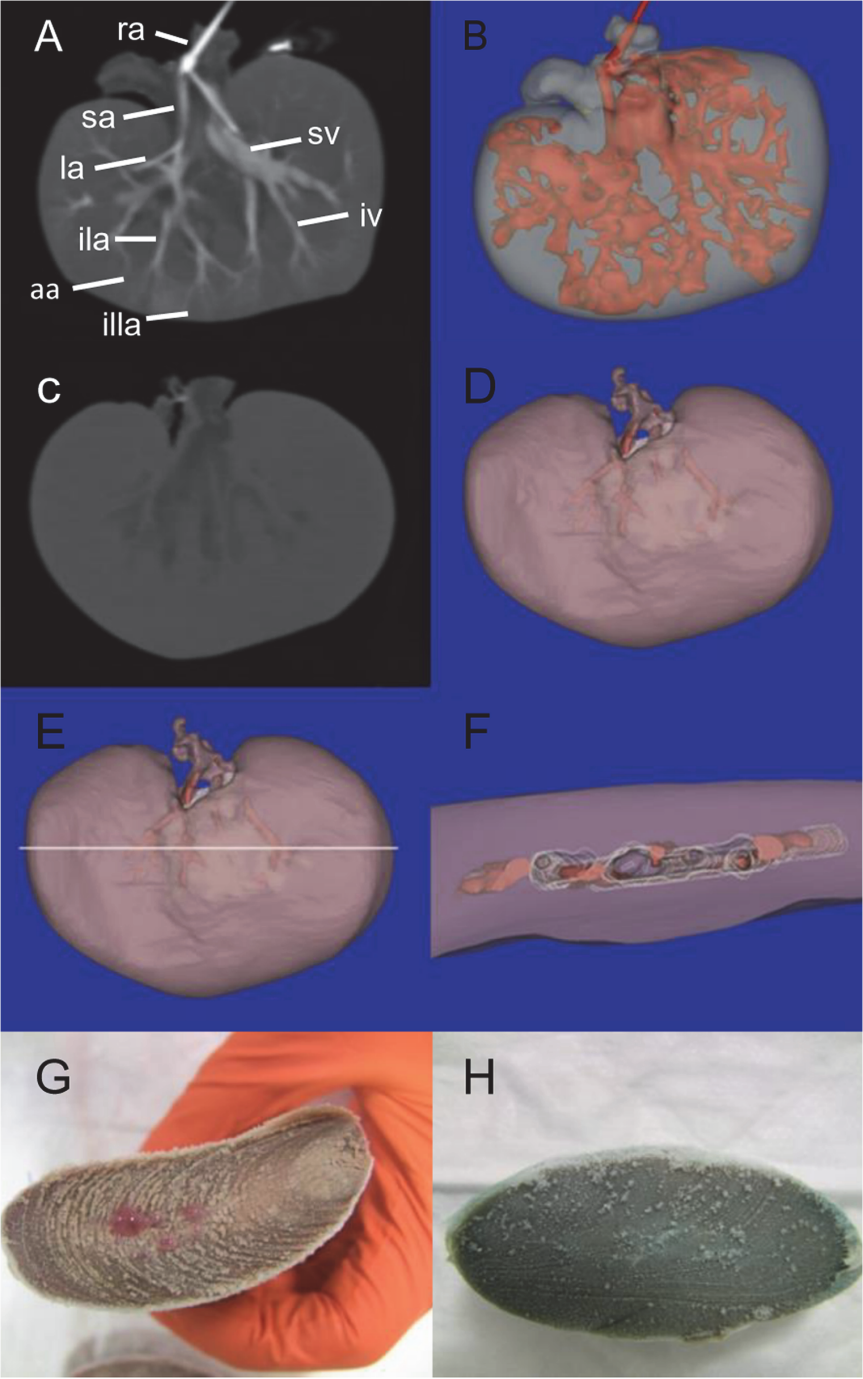
Contrast-enhanced reperfusion of Thiel embalmed and dehydrated pig kidneys with PP and diluted PEG. (A) Planar CT image shows two distinct areas of filling: the arterial and venous system (bright) demonstrating that PP recruits the major renal vessels and the renal tissue (dark grey). ra, renal artery; sa, segmental artery; la, lobar artery; ila, interlobar artery; aa, arcuate artery; illa, interlobular artery; iv, interlobar vein; sv, segmental vein. (B) Three-dimensional representation of the same kidney; red, arterial and venous system; transparent blue-grey, renal tissue showing less contrast. (C) Planar CT image illustrates one uniform area of filling: the arterial and venous system together with the renal tissue (light grey) and centrally a darker area (dark grey) representing the renal calices. (D) Three-dimensional representation of the same kidney. The renal tissue is transparent purple, and the segmental vessels are highlighted in red for illustration purposes. The central white polylined structure depicts the renal calices. (E) The white horizontal line represents the cross-sectional slice through the mid-central part of the kidney containing renal tissue, segmental vessels and the renal calices. (F) Top view of the caudal part following the virtual slicing illustrated in E. Transparent purple, renal tissue; the segmental vessels are highlighted in red and have the same contrast as the renal tissue; white polylines border the calices, which are not filled or are less filled by the contrast fluid. (G) Cross-section through frozen Thiel embalmed pig kidney reperfused with PP. Red PP is present in the major renal vessels. (H) Cross-section through frozen Thiel embalmed pig kidney reperfused with diluted PEG. Blue diluted PEG diffusely stains the sectioned renal surface.

CT-images showed PP spreading in the renal arterial tree up to the interlobular arteries without signs of extravasation. Later, drainage into the venous system occurred and clearly demonstrated PP running in the interlobar and segmental veins before eventually leaving the kidney via the renal vein. In contrast, an evenly renal distribution of diluted PEG was found making it impossible to distinguish major vessels from renal. parenchyma. Fig. 8 depicts the renal distributions of PP and diluted PEG.

**Fig 9:**
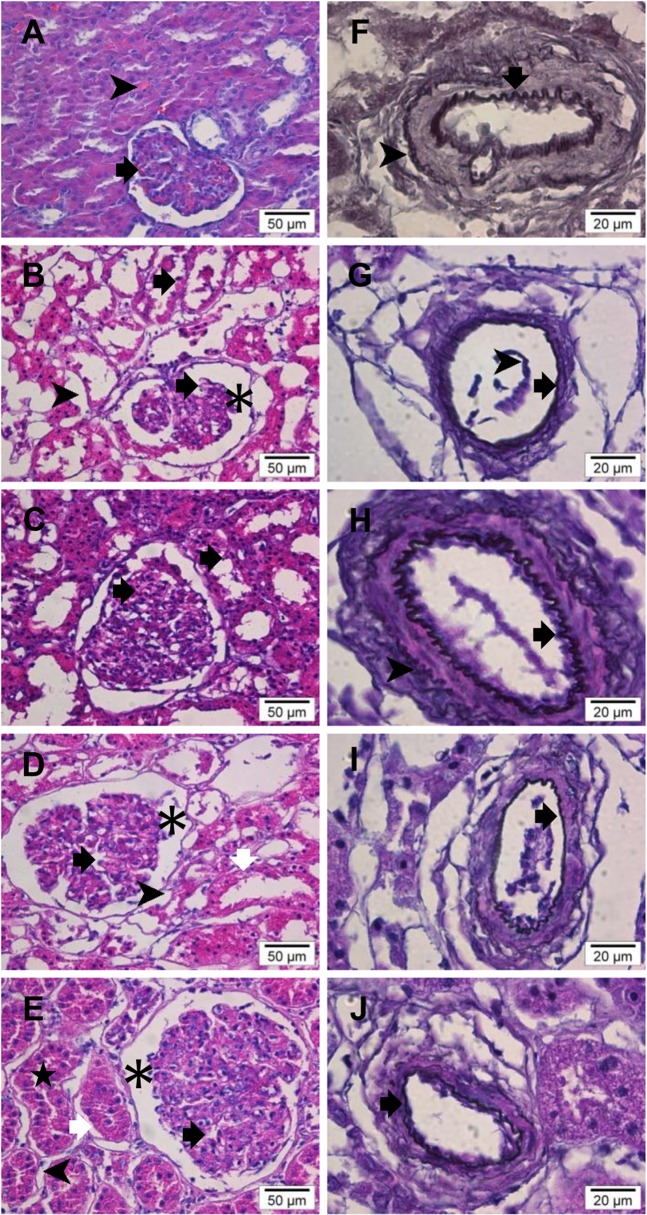
Morphological changes of renal cortex during embalming, dehydration and vascular reperfusion. Pig cadaver kidneys. A-E: Hematoxylin and Eosin staining (magnification: x 400). (A) Fresh renal cortex. Glomerular vessels (black arrow) and peritubular capillaries (arrowhead) contain red blood cells. (B) Thiel embalmed renal cortex. Cloudy cell swelling causes rupture of cells (black arrows) and glomerular disintegration. Embalming fluid accumulates in the interstitium (arrowhead) and Bowman’s space (asterisk). Red blood cells are absent. (C) Dehydrated Thiel embalmed renal cortex. Renal histology is similar to the fresh status. The glomeruli have a compact structure and cells have a dens appearance but their shape remains irregular (black arrows). There is no expansion of the interstitial space. (d) Reperfusion of Thiel embalmed dehydrated renal cortex with PP. Reperfusion causes dilation of the glomerular vessels (black arrow) and expansion of Bowman’s space (asterisk) and interstitial space (arrowhead). Tubular cells remain irregular and flat (white arrow) as in the dehydrated status suggesting that the perfusate does not interact with these cells. (e) Reperfusion of Thiel embalmed dehydrated renal cortex with diluted PEG. Glomerular vessels (arrow) and Bowman’s space (asterisk) are dilated. There is a fluid shift into the interstitial space (arrowhead). Widening of subepithelial space (white arrow) of enlarged tubular cells presumably due to uptake of the water component of diluted PEG. This causes narrowing of tubular lumina (star). F-J: Orcein staining (magnification: x 1000). (F) Fresh renal cortex. The wavy internal elastic lamina of arterioles is deep red brown (black arrow). The tunica adventitia is lightly stained (arrowhead). (G) Thiel embalmed renal cortex. More than half of the internal elastic lamina is flattened suggesting fluid accumulation in the vessel wall (black arrow). Partly detached endothelial cells (arrowhead). (H) Dehydrated Thiel embalmed renal cortex. Return of the wavy appearance of the internal (black arrow) and external (arrowhead) elastic membranes due to fluid loss. (I) Reperfusion of Thiel embalmed dehydrated renal cortex with PP. Partial flattening of the internal elastic lamina probably due to an increase of perfusate in the vessel wall (black arrow). (J) Reperfusion of Thiel embalmed dehydrated renal cortex with diluted PEG. The internal elastic lamina lost parts of its scalloped appearance which may be caused by a build-up of diluted PEG in the wall (black arrow).

The embalming procedure caused cell swelling and expansion of the interstitial space. Moreover, it effectively flushed remaining red blood cells and flattened the internal elastic lamina of arterioles. Subsequent dehydration removed the excess of fluid in every structural renal component. Prolonged reperfusion with both perfusates demonstrated glomerular vessels dilation, swelling of the interstitial space and partial flattening of the arteriolar internal elastic lamina. Reperfusion with PP did not result in significant structural changes of the tubular cells. In contrast, diffuse swelling of the tubular cells was observed after reperfusion with diluted PEG. Fig. 9 illustrates the morphological changes of renal tissue during embalming, dehydration and vascular reperfusion.

## Discussion

This study was the first to assess the vascular spreading of contrast-enhanced Thiel embalming solution and to determine its effect on the appearance of the original fresh tissue. We demonstrate that pig kidneys are a suitable model to evaluate this technique. Indeed, CT imaging showed that when a volume was injected as determined by Thiel (i.e., comparable to 18.170 kg for a human cadaver weighing 80 kg), there was a widespread distribution in the kidney without filling defects.

This distribution is feasible when embalming is done under controlled intra-arterial pressure using a pump. We emphasize that a pressure lower than 65 mmHg is essential to limit unnecessary vessel damage and ongoing local congestion of embalming product in the interstitial space. The mean arterial blood pressure in adult pigs is 60–65 mmHg (unpublished data). Despite controlling the pressure, local accumulation, interstitial space swelling, partial flattening of the arteriolar internal elastic membrane and significant increases in weight and volume were observed. These changes are likely due to the injection of too much embalming fluid, which eventually extravasates, and they are favoured by the low viscosity of the embalming solution. Note that the embalming fluid must perfuse the capillaries for effective preservation. Due to early postmortem autolysis, leakage into the interstitial space is probably unavoidable but must be minimized to avoid swelling and deformation. Thiel embalming fluid is very thin and is less viscous than blood (i.e., 4–5 mPas at body temperature), and therefore, it easily flows through the smallest vessels without generating high inlet pressures [15]. The use of the large volume of vascular embalming solution, as originally proposed by Thiel, must be questioned as it causes significant increases in weight and volume. During embalming, venous loss of this solution (i.e., transparent or serosanguinous venous drainage) was observed in 40% of the kidneys, which were, however, adequately preserved. It should be borne in mind that diminishing the embalming volume without hampering tissue preservation must be undertaken with caution. Consequently, dehydration of embalmed kidneys by salt water immersion can be a good solution. Notably, blood is still present in the vast majority of embalmed kidneys, suggesting that complete venous drainage of blood is superfluous. Notwithstanding its exploratory character, this study observed the course of the embalming fluid in intact vessels, making it impossible to assess if atherosclerosis affects the notable variation in the quality of embalmed tissue within and among Thiel cadavers (unpublished data). Other causes for this observation may be the varying periods between death and the initiation of embalming or deficiencies in the embalming properties of the Thiel fluid, which are unlikely.

Intriguingly, vascular perfusion alone effectively embalmed every kidney, enabling storage in the refrigerator for more than two months. This result could be expected because the CT images revealed no zones lacking embalming fluid. It is important to note that we did not immerse the kidneys in an embalming bath as recommended by Thiel. As extensively demonstrated by Thiel, immersion is essential to embalm the skin and subcutaneous tissue of human bodies, but immersion seems superfluous when preserving a kidney. As mentioned above, embalming causes swelling and deformation, so an appropriate and simple method was examined to re-establish the original weight of the embalmed kidneys in a fast, controlled manner. Embalming-induced weight and volume gains can be successfully reduced by immersion in a concentrated salt solution. Shrinkage of the interstitial space and return of the original wavy structure of the internal and external elastic membranes illustrate this fluid loss. This was the first report showing that Thiel embalmed organs can be quickly dehydrated under controlled circumstances to reinstate their original status. Note that the majority of kidneys are obviously shrunken and discoloured due to blood loss but remain pliable. Our intention to reduce the weight of embalmed tissue is part of a wider project on blood-like vascular reperfusion of Thiel embalmed human cadavers. Therefore, a continuous pump-driven flow mimicking blood circulation was tested in the vessels of dehydrated Thiel embalmed kidneys. We clearly demonstrate that both PP and diluted PEG effectively circulate in this kidney model. CT imaging confirms they had a widespread renal distribution without filling defects. The results indicated that PP clearly fills the major vessels and renal tissue, whereas diluted PEG more diffusely spreads in the kidney. Certainly, PP is superior because no significant weight or volume gain was observed after more than 60 minutes of reperfusion. Moreover, PP generates lower pressures and does not influence the pliability and appearance of the organs. Indeed, there is some expansion of the interstitial space and partial flattening of the internal elastic lamina of the arterioles suggesting extravasation, but the major reason why lower pressures are observed is the absence of structural tubular changes during reperfusion with PP. As PP is osmotically inactive, it does not interfere with the tubular cells, which remain flat. In contrast, we observed ongoing weight and volume gain and pressure increase during prolonged reperfusion with diluted PEG. This observation can be explained by cellular uptake of the water component causing diffuse swelling of the tubules across the renal parenchyma. Note that we encountered a gradual loss of fluid from the organ into the environment during storage. Probably, this is the water component of the diluted PEG, which leaves the kidney and may permit a second reperfusion. Researchers previously reperfused animal models with several types of perfusates [16–18]. However, these experiments were performed on fresh tissue.

Hence, it is likely that combining embalming and dehydration will enable reperfusion of Thiel embalmed human cadavers. In our experience, embalming-induced swelling without subsequent dehydration hindered pump-driven vascular reperfusion in an adult pig (unpublished data). Interestingly, a long immersion of a Thiel embalmed pig in a concentrated salt solution caused significant weight loss (unpublished data), which may allow for vascular reperfusion with PP. Thus, future research should focus on validating this model in an embalmed and dehydrated organ system or total body.

## Conclusions

The Thiel embalming procedure is a complex process, and several essential steps must be fulfilled for adequate preservation. We show that Thiel embalming fluid has a low viscosity and therefore easily flows in intact vessels and diffusely spreads in a kidney model. As such, other factors may contribute to the observed variation in tissue preservation within and among Thiel embalmed cadavers. Further, it is recommended that this mixture is administered under physiological pressure using a pump to limit vascular damage and needless subsequent extravasation that later escapes from the body. We posit that injecting a larger volume to enhance the embalming quality will result in unnecessary swelling and deformation, and we underscore that the recommended embalming volume is sufficient, but useless congestion occurs. Immersion in a concentrated salt solution is a quick, easy and controlled method to remove accumulated fluid and re-establish the organs’ original status without jeopardizing the embalming quality. The present study provides a useful basis for assessing the course of the embalming mixture in human bodies, for restoring the cadavers’ initial appearance and for broadening our knowledge about the embalming procedure. Furthermore, this model seems ideal to establish prolonged vascular reperfusion in Thiel embalmed cadavers by mimicking lifelike circumstances to learn human anatomy, to test new devices and to practice surgical procedures.

## Supporting Information

S1 Dataset Effect of vascular embalming on the macroscopic appearance of the kidney (experiment 1). (XLSX)

S2 Dataset Statistical analysis of experiment 1. (DOCX)

S3 Dataset Calculation of the dynamic viscosity of Thiel embalming solution. (XLSX)

S4 Dataset Embalming and vascular reperfusion of the kidney (experiment 2). (XLSX)

S5 Dataset Statistical analysis of experiment 2. (DOC)

S6 Dataset Effect of vascular reperfusion on renal pliability, swelling, and capsular leakage (experiment 2). (XLSX)
