# Supplementary material for: Understanding Thiel Embalming in Pig Kidneys to Develop a New Circulation Model
Source: PLoS One. 2015 Mar 25;10(3):e0120114. doi: 10.1371/journal.pone.0120114 (PMC4373718; doi:10.1371/journal.pone.0120114)
Supplement: S2 Dataset — (DOCX) [file pone.0120114.s002.docx]

**One Way Repeated Measures Analysis of Variance** vrijdag, september 06, 2013, 10:46:08

**Data source:** Data 1 in Percentage weight change embalming and dehydration 15 pig kidneys.JNB

**Normality Test (Shapiro-Wilk)** Passed (P = 0,300)

**Equal Variance Test:** Failed (P < 0,050)

Test execution ended by user request, RM ANOVA on Ranks begun

**Friedman Repeated Measures Analysis of Variance on Ranks** vrijdag, september 06, 2013, 10:46:08

**Data source:** Data 1 in Percentage weight change embalming and dehydration 15 pig kidneys.JNB

**Group N Missing Median 25% 75%**

Col 1 15 0 13,578 11,705 16,003

Col 2 15 0 -13,663 -27,302 -8,834

Col 3 15 0 1,000 1,000 1,000

Chi-square= 30,000 with 2 degrees of freedom. (P = <0,001)

The differences in the median values among the treatment groups are greater than would be expected by chance; there is a statistically significant difference (P = <0,001)

To isolate the group or groups that differ from the others use a multiple comparison procedure.

All Pairwise Multiple Comparison Procedures (Tukey Test):

**Comparison Diff of Ranks q P<0,05**

Col 1 vs Col 2 30,000 7,746 Yes

Col 1 vs Col 3 15,000 3,873 Yes

Col 3 vs Col 2 15,000 3,873 Yes

Note: The multiple comparisons on ranks do not include an adjustment for ties.
