# Supplementary material for: Understanding Thiel Embalming in Pig Kidneys to Develop a New Circulation Model
Source: PLoS One. 2015 Mar 25;10(3):e0120114. doi: 10.1371/journal.pone.0120114 (PMC4373718; doi:10.1371/journal.pone.0120114)
Supplement: S5 Dataset — (DOC) [file pone.0120114.s005.doc]

# Balseming en Reperfusie van 20 nieren met PEG / PP

Afkortingen:

WAD weight after dissection

WAE weight after embalming

W7E weight after 7 days brining

WSR weight at start reperfusion

WVD weight at veinage drainage

WSE weight after 60 minutes of reperfusion

WHE weight after 120 minutes of reperfusion

VAD volume after dissection

VAE volume after embalming

V7E volume after 7 days brining

VSR volume at start reperfusion

VVD volume at venous drainage

VSE volume after 60 minutes of reperfusion

VHE volume after 120 minutes of reperfusion

Five, Ten, Fifteen, Etc. aantal minute na start reperfusie waarop drukmeting genoteerd

De Friedmantest wordt gebruikt voor de vergelijkende analyse van de verkregen data bij elke nier over het verloop van de tijd. De Wilcoxon matched pairs signed ranks test wordt gebruikt voor de vergelijkende analyse van de data om de resultaten van de Friedmantest te verduidelijken (waar bevindt het significant verschil zich?) Dit gebruik makend van SPSS 21.0. Het betrouwbaarheidsintervan is 95% (p-waarde: p<0.05)

**Friedman test** Friedman test wordt gebruikt voor een longitudinale studie. Herhaalde waarnemingen van dezelfde variabele bij eenzelfde individu (de nier) en onder dezelfde experimentele voorwaarden, kunnen bij het vergelijken van groepen niet geanalyseerd worden als onafhankelijke waarnemingen. Wanneer het aantal waarnemingen hetzelfde is voor alle individuen (geen ontbrekende waarnemingen), dan kan men beroep doen op een variantieanalysetechniek voor herhaalde waarnemingen (repeated measurements)
 De Friedman test is een niet-parametrische test voor een enkelvoudige variantieanalyse voor herhaalde waarnemingen. Deze test wordt gebruikt wanneer de voorwaarden voor het gebruik van de parametrische test niet vervuld zijn of wanneer de studievariabele van het ordinale type is.
 In tegenstelling evenwel tot het parametrische complexe model dat toelaat gepaarde metingen te vergelijken tussen twee groepskenmerken en/of bepaalde covariabelen in rekening te brengen, kan de Friedman-test slechts gebruikt worden voor een enkelvoudige dataset waarbij men beschikt over een steekproef van n individuen waarbij men herhaalde metingen heeft bekomen in k meetsituaties.
 Stel dat men beschikt over een enkelvoudige gegevenstabel met k herhaalde mtingen (zijnde tijdstippen of andere meetomstandigheden):
 De metingen die herhaald verkregen werden voor de k meetmomenten worden simpelweg vervangen door hun rangorde in de rij. Rangen worden toegekend van 1 tot en met k. Bij gelijke waarde wordt daar op de gebruikelijke manier mee omgesprongen door deze te vervangen door de gemiddelde rang. Vervolgens zal men alle rangen per kolom optellen en aldus de rangsommen verkrijgen.
 Aan de hand van de rangsommen wordt vervolgens de statistische grootheid berekend. Deze statistische grootheid beantwoordt aan de chi-kwaraatverdeling.
 Bij een significant resultaat zegt deze Friedman-test op zichzelf niet waar (tussen welke meetmomenten) het significante verschil zich afspeelt. Dit inzicht wordt geboden door twee-à-twee gepaarde Wilcoxon-testen uit te voeren met correctie voor het meervoudig testen (Bonferroni).
 De Friedman-test is de onmiddellijke uitbreiding van de gepaarde Wilcoxon-test.
 Indien de steekproefgrootte het toelaat kan men de gegevens grafisch voorstellen met behulp van een lijndiagram.

**Continue variabelen – niet-parametrisch – gepaarde Wilcoxon-test** Deze test wordt gebruikt om twee gepaarde steekproeven met elkaar te vergelijken in het geval van continue variabelen. Gepaarde betekent dat de studievariabele bij elk individu verkregen werd onder twee omstandigheden. Een statistische test voor het vergelijken van gepaarde gegevens is gebaseerd op het verschil tussen de gepaarde waarnemingen. In feite wordt hierdoor het probleem herleid tot de situatie waarbij men beschikt over één steekproef van de verschillen.
 Deze test is in het bijzonder aangewezen indien aan de voorwaarde dat het verschil tussen de paren onderling normaald verdeel moeten zijn niet voldaan is.
 De geijkte naam voor deze test is de Wilcoxon matched-pairs signed-ranks-test, dit is de tegenhanger van de parametrische gepaarde Student’s t-test.
Bron: Inleiding ot de biomedische statistiek. Georges De Moor en Georges Van Maele. Acco.

## Gewicht (20 nieren)

**Beschrijvende statistiek**

| **Descriptive Statistics** | | | | | | |
| --- | --- | --- | --- | --- | --- | --- |
|  | N | Range | Minimum | Maximum | Mean | Std. Deviation |
| WAD | 20 | 108,32 | 121,97 | 230,29 | 157,1800 | 23,81241 |
| WAE | 20 | 126,42 | 139,02 | 265,44 | 180,8395 | 28,07216 |
| W7E | 20 | 86,39 | 108,99 | 195,38 | 142,3715 | 21,46389 |
| Valid N (listwise) | 20 |  |  |  |  |  |

WAD weight after dissection of the kidney
WAE weight after embalming
W7E weight after 7 days brining

**Friedman test**

WAD – WAE – W7E

| **Test Statistics^a^** | |
| --- | --- |
| N | 20 |
| Chi-Square | 38,100 |
| df | 2 |
| Asymp. Sig. | ,000 |

Wilcoxon test

| **Test Statistics^a^** | | | |
| --- | --- | --- | --- |
|  | WAE - WAD | W7E - WAD | W7E - WAE |
| Z | -3,921^b^ | -3,883^c^ | -3,920^c^ |
| Asymp. Sig. (2-tailed) | ,000 | ,000 | ,000 |

**Besluit**

Er is een statistische significant verschil tussen het gewicht gemeten na dissectie, na balseming en na 7 dagen ‘brining’.

(zie grafiek 1)

## Volume (20 nieren)

Beschrijvende statistiek

| **Descriptive Statistics** | | | | | | |
| --- | --- | --- | --- | --- | --- | --- |
|  | N | Range | Minimum | Maximum | Mean | Std. Deviation |
| VAD | 20 | 98,00 | 118,00 | 216,00 | 152,2000 | 28,12117 |
| VAE | 20 | 117,00 | 127,00 | 244,00 | 170,1000 | 27,78470 |
| V7E | 20 | 85,00 | 96,00 | 181,00 | 123,6000 | 21,06231 |
| Valid N (listwise) | 20 |  |  |  |  |  |

**Friedman test**

VAD – VAE – V7E

| **Test Statistics^a^** | |
| --- | --- |
| N | 20 |
| Chi-Square | 38,100 |
| df | 2 |
| Asymp. Sig. | ,000 |

Wilcoxon test

| **Test Statistics^a^** | | | |
| --- | --- | --- | --- |
|  | VAE - VAD | V7E - VAD | V7E - VAE |
| Z | -3,923^b^ | -3,772^c^ | -3,922^c^ |
| Asymp. Sig. (2-tailed) | ,000 | ,000 | ,000 |

**Besluit**

Er is een statistische significant verschil tussen het volume gemeten na dissectie, na balseming en na 7 dagen ‘brining’.

(zie grafiek 2)

OPMERKING:

Gewicht en Volume ‘at start reperfusion’ opnemen of niet? (groot verschil in ‘droogperiode’)

## PARAFFINE

**Beschrijvende statistiek gewicht en volume**

| **Descriptive Statistics** | | | | | | |
| --- | --- | --- | --- | --- | --- | --- |
|  | N | Range | Minimum | Maximum | Mean | Std. Deviation |
| WAD | 10 | 108,32 | 121,97 | 230,29 | 158,6540 | 31,53669 |
| VAD | 10 | 98,00 | 118,00 | 216,00 | 148,8000 | 29,80231 |
| WAE | 10 | 126,42 | 139,02 | 265,44 | 182,5040 | 37,62767 |
| VAE | 10 | 117,00 | 127,00 | 244,00 | 167,3000 | 33,41008 |
| W7E | 10 | 86,39 | 108,99 | 195,38 | 145,2840 | 28,56689 |
| V7E | 10 | 85,00 | 96,00 | 181,00 | 127,4000 | 27,28532 |
| WSR | 10 | 86,21 | 109,01 | 195,22 | 143,1760 | 29,52167 |
| VSR | 10 | 78,00 | 96,00 | 174,00 | 124,6000 | 26,62163 |
| WVD | 10 | 90,67 | 111,24 | 201,91 | 147,4810 | 30,20975 |
| VVD | 10 | 84,00 | 96,00 | 180,00 | 127,5000 | 28,50049 |
| WSE | 10 | 95,62 | 113,38 | 209,00 | 152,0160 | 30,94268 |
| VSE | 10 | 82,00 | 99,00 | 181,00 | 132,1000 | 28,98448 |
| WHE | 10 | 95,33 | 113,67 | 209,00 | 152,3990 | 31,36672 |
| VHE | 10 | 82,00 | 99,00 | 181,00 | 131,9000 | 30,97831 |
| Valid N (listwise) | 10 |  |  |  |  |  |

**Gewicht**

**Friedman test**

WAD – WAE – W7E – WSR – WVD – WSE - WHE

| N | 10 |
| --- | --- |
| Chi-Square | 52,798 |
| df | 6 |
| Asymp. Sig. | ,000 |

WSR – WVD – WSE - WHE

| N | 10 |
| --- | --- |
| Chi-Square | 27,545 |
| df | 3 |
| Asymp. Sig. | ,000 |

WVD – WSE - WHE

| N | 10 |
| --- | --- |
| Chi-Square | 27,545 |
| df | 3 |
| Asymp. Sig. | ,000 |

Wilcoxon test

|  | | WAE - WAD | W7E - WAD | WSR - WAD | WVD - WAD | WSE - WAD | WHE - WAD | W7E - WAE | WSR - WAE | WVD - WAE | WSE - WAE | WHE - WAE | WSR - W7E | WVD - W7E | WSE - W7E | WHE - W7E | WVD - WSR | WSE - WSR | WHE - WSR | WSE - WVD | WHE - WVD | WHE - WSE |
| --- | --- | --- | --- | --- | --- | --- | --- | --- | --- | --- | --- | --- | --- | --- | --- | --- | --- | --- | --- | --- | --- | --- |
| Z | -2,803^b^ | | -2,701^c^ | -2,803^c^ | -2,701^c^ | -2,090^c^ | -1,784^c^ | -2,803^c^ | -2,803^c^ | -2,803^c^ | -2,803^c^ | -2,803^c^ | -2,090^c^ | -1,988^b^ | -2,803^b^ | -2,803^b^ | -2,803^b^ | -2,803^b^ | -2,803^b^ | -2,803^b^ | -2,803^b^ | -1,125^b^ |
| Asymp. Sig. (2-tailed) | ,005 | | ,007 | ,005 | ,007 | ,037 | ,074 | ,005 | ,005 | ,005 | ,005 | ,005 | ,037 | ,047 | ,005 | ,005 | ,005 | ,005 | ,005 | ,005 | ,005 | ,260 |

**Besluit**

Er is een significant verschil tussen de gemeten gewichten startend van het gewicht na dissectie, het gewicht bij start reperfusie en gewicht na veneuze drainage.

Dit significant verschil bevindt zich **niet** tussen de paren:
gewicht na dissectie – gewicht na 60 minuten reperfusie
gewicht na dissectie – gewicht na 120 minuten reperfusie
gewicht na 7 dagen brining – gewicht bij start reperfusie
gewicht na 7 dagen brining – gewicht na veneuze drainage
gewicht na 60 minuten reperfusie – gewicht na 120 minuten reperfusie

(zie grafiek 3)

**Volume**

**Friedman test**

VAD – VAE – V7E – VSR – VVD – VSE - VHE

| N | 10 |
| --- | --- |
| Chi-Square | 45,941 |
| df | 6 |
| Asymp. Sig. | ,000 |

VSR – VVD – VSE – VHE

| N | 10 |
| --- | --- |
| Chi-Square | 19,659 |
| df | 3 |
| Asymp. Sig. | ,000 |

VVD VSE – VHE

| N | 10 |
| --- | --- |
| Chi-Square | 13,067 |
| df | 2 |
| Asymp. Sig. | ,001 |

Wilcoxon test

| **Test Statistics^a^** | | | | | | | | | | | | | | | | | | | | | |
| --- | --- | --- | --- | --- | --- | --- | --- | --- | --- | --- | --- | --- | --- | --- | --- | --- | --- | --- | --- | --- | --- |
|  | VAE - VAD | V7E - VAD | VSR - VAD | VVD - VAD | VSE - VAD | VHE - VAD | V7E - VAE | VSR - VAE | VVD - VAE | VSE - VAE | VHE - VAE | VSR - V7E | VVD - V7E | VSE - V7E | VHE - V7E | VVD - VSR | VSE - VSR | VHE - VSR | VSE - VVD | VHE - VVD | VHE - VSE |
| Z | -2,807^b^ | -2,601^c^ | -2,803^c^ | -2,805^c^ | -2,805^c^ | -2,601^c^ | -2,807^c^ | -2,803^c^ | -2,805^c^ | -2,807^c^ | -2,807^c^ | -,593^c^ | -,059^b^ | -1,123^b^ | -,714^b^ | -1,970^b^ | -2,668^b^ | -2,547^b^ | -2,675^b^ | -2,374^b^ | -,272^c^ |
| Asymp. Sig. (2-tailed) | ,005 | ,009 | ,005 | ,005 | ,005 | ,009 | ,005 | ,005 | ,005 | ,005 | ,005 | ,553 | ,953 | ,262 | ,475 | ,049 | ,008 | ,011 | ,007 | ,018 | ,785 |

**Besluit**

Er is een significant verschil tussen de gemeten volumes startend van het volume na dissectie, het volume bij start reperfusie en volume na veneuze drainage.

Dit significant verschil bevindt zich **niet** tussen de paren:
volume na 7 dagen brining – volume bij start reperfusie
volume na 7 dagen brining – volume na veneuze drainage
volume na 7 dagen brining – volume na 60 minuten reperfusie
volume na 7 dagen brining – volumena 120 minuten reperfusie
volume na 60 minuten reperfusie – volumena 120 minuten reperfusie

(zie grafiek 4)

**Drukmeting**

**Friedman test**

0 – 60 minuten

| **Test Statistics^a^** | |
| --- | --- |
| N | 10 |
| Chi-Square | 58,704 |
| df | 11 |
| Asymp. Sig. | ,000 |

60 – 120 minuten

| **Test Statistics^a^** | |
| --- | --- |
| N | 10 |
| Chi-Square | 21,178 |
| df | 11 |
| Asymp. Sig. | ,032 |

0-120 minuten

| **Test Statistics^a^** | |
| --- | --- |
| N | 10 |
| Chi-Square | 64,576 |
| df | 23 |
| Asymp. Sig. | ,000 |

**Besluit**

Er is een statistisch significant drukverschil over 2h reperfusie.

(zie grafiek 5)

## PEG/H2O

**Gewicht en Volume**

Beschrijvende statistiek

| **Descriptive Statistics** | | | | | | |
| --- | --- | --- | --- | --- | --- | --- |
|  | N | Range | Minimum | Maximum | Mean | Std. Deviation |
| WAD | 10 | 50,55 | 135,31 | 185,86 | 155,7060 | 14,05966 |
| VAD | 10 | 74,00 | 118,00 | 192,00 | 155,6000 | 27,48818 |
| WAE | 10 | 46,96 | 158,26 | 205,22 | 179,1750 | 15,54524 |
| VAE | 10 | 62,00 | 146,00 | 208,00 | 172,9000 | 22,27330 |
| W7E | 10 | 31,45 | 119,96 | 151,41 | 139,4590 | 11,73322 |
| V7E | 10 | 36,00 | 100,00 | 136,00 | 119,8000 | 12,64735 |
| WSR | 10 | 31,98 | 121,70 | 153,68 | 138,6110 | 11,76255 |
| VSR | 10 | 30,00 | 104,00 | 134,00 | 115,3000 | 10,40353 |
| WVD | 10 | 43,10 | 133,65 | 176,75 | 153,4550 | 14,49652 |
| VVD | 10 | 39,00 | 112,00 | 151,00 | 132,8000 | 12,64735 |
| WSE | 10 | 73,76 | 136,74 | 210,50 | 178,5510 | 21,23147 |
| VSE | 10 | 73,00 | 116,00 | 189,00 | 157,3000 | 22,96882 |
| WHE | 10 | 86,26 | 139,31 | 225,57 | 191,5520 | 26,73239 |
| VHE | 10 | 87,00 | 117,00 | 204,00 | 167,7000 | 25,78565 |
| Valid N (listwise) | 10 |  |  |  |  |  |

**Gewicht**

**Friedman**

WAD – WAE – W7E – WSR – WVD – WSE – WHE

| **Test Statistics^a^** | |
| --- | --- |
| N | 10 |
| Chi-Square | 48,643 |
| df | 6 |
| Asymp. Sig. | ,000 |

WSR – WVD – WSE – WHE

| **Test Statistics^a^** | |
| --- | --- |
| N | 10 |
| Chi-Square | 28,920 |
| df | 3 |
| Asymp. Sig. | ,000 |

WVD – WSE – WHE

| **Test Statistics^a^** | |
| --- | --- |
| N | 10 |
| Chi-Square | 20,000 |
| df | 2 |
| Asymp. Sig. | ,000 |

Wilcoxon

|  | WAE - WAD | W7E - WAD | WSR - WAD | WVD - WAD | WSE - WAD | WHE - WAD | W7E - WAE | WSR - WAE | WVD - WAE | WSE - WAE | WHE - WAE | WSR - W7E | WVD - W7E | WSE - W7E | WHE - W7E | WVD - WSR | WSE - WSR | WHE - WSR | WSE - WVD | WHE - WVD | WHE - WSE |
| --- | --- | --- | --- | --- | --- | --- | --- | --- | --- | --- | --- | --- | --- | --- | --- | --- | --- | --- | --- | --- | --- |
| Z | -2,805^b^ | -2,805^c^ | -2,805^c^ | -,969^c^ | -2,601^b^ | -2,601^b^ | -2,805^c^ | -2,805^c^ | -2,805^c^ | -,357^b^ | -1,071^b^ | -,561^b^ | -2,499^b^ | -2,601^b^ | -2,703^b^ | -2,703^b^ | -2,805^b^ | -2,805^b^ | -2,805^b^ | -2,805^b^ | -2,805^b^ |
| Asymp. Sig. (2-tailed) | ,005 | ,005 | ,005 | ,333 | ,009 | ,009 | ,005 | ,005 | ,005 | ,721 | ,284 | ,575 | ,012 | ,009 | ,007 | ,007 | ,005 | ,005 | ,005 | ,005 | ,005 |

**Besluit**

Er is een significant verschil tussen de gemeten gewichten startend van het gewicht na dissectie, het gewicht bij start reperfusie en gewicht na veneuze drainage.

Dit significant verschil bevindt zich **niet** tussen de paren:
gewicht na dissectie - gewicht na veneuze drainage
gewicht na balseming – gewicht na 60 minuten reperfusie
gewicht na balseming – gewicht na 120 minuten reperfusie
gewicht na 7 dagen brining – gewicht bij start reperfusie

(zie grafiek 6)

**Volume**

**Friedman**

VAD – VAE – V7E – VSR – VVD – VSE – VHE

| **Test Statistics^a^** | |
| --- | --- |
| N | 10 |
| Chi-Square | 44,297 |
| df | 6 |
| Asymp. Sig. | ,000 |

VSR – VVD – VSE – VHE

| **Test Statistics^a^** | |
| --- | --- |
| N | 10 |
| Chi-Square | 28,920 |
| df | 3 |
| Asymp. Sig. | ,000 |

VVD – VSE – VHE

| **Test Statistics^a^** | |
| --- | --- |
| N | 10 |
| Chi-Square | 18,200 |
| df | 2 |
| Asymp. Sig. | ,000 |

Wilcoxon

| **Test Statistics^a^** | | | | | | | | | | | | | | | | | | | | | |
| --- | --- | --- | --- | --- | --- | --- | --- | --- | --- | --- | --- | --- | --- | --- | --- | --- | --- | --- | --- | --- | --- |
|  | VAE - VAD | V7E - VAD | VSR - VAD | VVD - VAD | VSE - VAD | VHE - VAD | V7E - VAE | VSR - VAE | VVD - VAE | VSE - VAE | VHE - VAE | VSR - V7E | VVD - V7E | VSE - V7E | VHE - V7E | VVD - VSR | VSE - VSR | VHE - VSR | VSE - VVD | VHE - VVD | VHE - VSE |
| Z | -2,810^b^ | -2,805^c^ | -2,805^c^ | -2,091^c^ | -,255^c^ | -1,072^b^ | -2,807^c^ | -2,812^c^ | -2,805^c^ | -1,429^c^ | -,817^c^ | -1,129^c^ | -2,196^b^ | -2,603^b^ | -2,601^b^ | -2,807^b^ | -2,807^b^ | -2,812^b^ | -2,805^b^ | -2,810^b^ | -2,148^b^ |
| Asymp. Sig. (2-tailed) | ,005 | ,005 | ,005 | ,037 | ,799 | ,284 | ,005 | ,005 | ,005 | ,153 | ,414 | ,259 | ,028 | ,009 | ,009 | ,005 | ,005 | ,005 | ,005 | ,005 | ,032 |

**Besluit**

Er is een significant verschil tussen de gemeten volumes startend van het volume na dissectie, het volume bij start reperfusie en gewicht na veneuze drainage.

Dit significant verschil bevindt zich **niet** tussen de paren:
volume na dissectie – volumena 60 minuten reperfusie
volume na dissectie – volume na 120 minuten reperfusie
volume na balseming – volume na 60 minuten reperfusie
volume na balseming – volume na 120 minuten reperfusie
volume na 7 dagen brining – volume bij start reperfusie

(zie grafiek 7)

**Drukmeting**

**Friedman**

0 – 60 minuten

| **Test Statistics^a^** | |
| --- | --- |
| N | 9 |
| Chi-Square | 19,290 |
| df | 11 |
| Asymp. Sig. | ,056 |

65 – 120 minuten

| **Test Statistics^a^** | |
| --- | --- |
| N | 10 |
| Chi-Square | 49,159 |
| df | 11 |
| Asymp. Sig. | ,000 |

0 – 120 minuten

| **Test Statistics^a^** | |
| --- | --- |
| N | 9 |
| Chi-Square | 75,172 |
| df | 23 |
| Asymp. Sig. | ,000 |

**Besluit**

Er is een statistisch significant verschil in druk over de 2h.
Opgesplitst is er geen significant verschil in druk in het 1^e^ uur, maar wel in het 2^e^ uur van de reperfusie.

( zie grafiek 8)
